# Auto-inhibitory Mechanism of the Human Mitochondrial RNase P Protein Complex

**DOI:** 10.1038/srep09878

**Published:** 2015-04-30

**Authors:** Fengzhi Li, Xiaofen Liu, Weihong Zhou, Xue Yang, Yuequan Shen

**Affiliations:** 1 State Key Laboratory of Medicinal Chemical Biology, Nankai University, 94 Weijin Road, Tianjin 300071, China; 2 College of Life Sciences, Nankai University, 94 Weijin Road, Tianjin 300071, China; 3 Synergetic Innovation Center of Chemical Science and Engineering, 94 Weijin Road, Tianjin 300071, China

## Abstract

It is known that tRNAs play an essential role in genetic information transfer from DNA to protein. The maturation of tRNA precursors is performed by the endoribonuclease RNase P, which classically consists of a main RNA segment and accessory proteins. However, the newly identified human mitochondrial RNase P-like protein (MRPP123) complex is unique in that it is composed of three proteins without RNA. Here, we determined the crystal structure of MRPP123 complex subunit 3 (MRPP3), which is thought to carry out the catalytic reaction. A detailed structural analysis in combination with biochemical assays suggests that MRPP3 is in an auto-inhibitory conformation in which metal ions that are essential for catalysis are excluded from the active site. Our results indicate that further regulation is necessary to rearrange the conformation of the active site of MRPP3 and trigger it, thus providing important information to understand the activation of MRPP123.

tRNAs serve as adaptors in translating nucleotide sequences into amino acid sequences and are usually transcribed as pre-tRNAs^1^,^2^,^3^. To reach their mature functional state, several steps of processing and modification are required^4^. This processing includes an initial cleavage reaction at both the 5′ and 3′ ends of the tRNA by the endoribonuclease RNase P and tRNase Z, respectively, followed by the action of nucleotidyl transferase that generates a matured tRNA carrying a CCA at its 3′ end. The endoribonuclease RNase P is a metallonuclease that hydrolyzes 5′ leader sequences from the pre-tRNA in the presence of Mg^2+^ or other divalent metal ions^2^,^5^. RNase P exists in almost all forms of life and is composed of RNA plus one or more proteins^6^. Among these components, the RNA is the catalytic subunit, and it recognizes the pre-tRNA and hydrolyzes it, resulting in the production of a 5′-RNA leader sequence with a 3′-OH and a 3′-tRNA with a 5′ phosphate^1^,^7^. The RNA subunit requires at least two divalent metal ions to participate in the chemistry of cleavage^6^,^8^. The protein subunits play important roles in catalysis and substrate recognition^9^,^10^. It has been shown that the number of assisting proteins increases with the complexity of the organism: one in bacteria, four or more in Archaea and at least nine in Eukarya^10^,^11^,^12^.

However, a new type of protein-only RNase P-like endoribonuclease called PRORP (proteinaceous RNase P) was recently found in human mitochondria and spinach chloroplasts and devoid of a catalytic RNA component^13^,^14^,^15^. Moreover, the crystal structure of PRORP1 was recently solved^8^,^16^. It contains five tandem PPR motifs, a central domain and a metallonuclease domain. PRORP1 catalyzes the 5′ end of pre-tRNA using a two-metal-ion mechanism, and the activity is higher with Mg^2+^ and Mn^2+^ compared with Ca^2+^ or Zn^2+^. Interestingly, PRORP1 can functionally substitute for *Escherichia coli* RNase P *in vivo*^3^,^15^,^17^.

In human mitochondria, the protein-only RNase P-like endoribonuclease (named MRPP123 hereafter) complex is composed of three proteins, MRPP1, MRPP2 and MRPP3^18^. MRPP1 belongs to the TRMT10 family^19^ and may be involved in the methylation of G9 in human mitochondrial tRNA. However, the methylation of G9 is not necessary for removal of the tRNA 5′ leader^20^. The role of MRPP1 in human mitochondrial tRNA processing awaits further investigation. MRPP2 is a multifunctional protein that is a member of the short-chain dehydrogenase family^21^,^22^; however, its dehydrogenase activity is not couple with its role in the endonuclease^20^. Additionally, MRPP2 binds to amyloid-β with high affinity, suggesting potential involvement in the pathogenesis of Alzheimer's disease^23^. The functional role of MRPP2 in the MRPP123 complex has remained unknown until now.

MRPP3 is the third member in the MRPP123 complex, and it possibly acts as the catalytic subunit of MRPP123^1^,^18^. It appears to loosely connect with recombinant MRPP1 and MRPP2 proteins in a particularly salt-dependent interaction^18^. MRPP3 contains three domains, a PPR domain, which is implicated in RNA binding, a metallonuclease domain and a central domain. MRPP3 is homologous with PRORP1, which exists in the mitochondria and chloroplasts of *Arabidopsis thaliana*^15^*.* However, MRPP3 fails to cleave the 5′ sequence of mitochondrial pre-RNA in the absence of MRPP1 and MRPP2, whereas PRORP1 can carry out this cleavage step by itself^18^.

To understand how the MRPP123 complex carries out its catalytic function, we report for the first time the crystal structure of the metallonuclease domain plus the central domain of human MRPP3 (named MRPP3-C hereafter, residues 274–583). We found that the active site of MRPP3-C retains an auto-inhibitory conformational state.

## Results

### Overall structure of MRPP3-C

**Figure 1:**
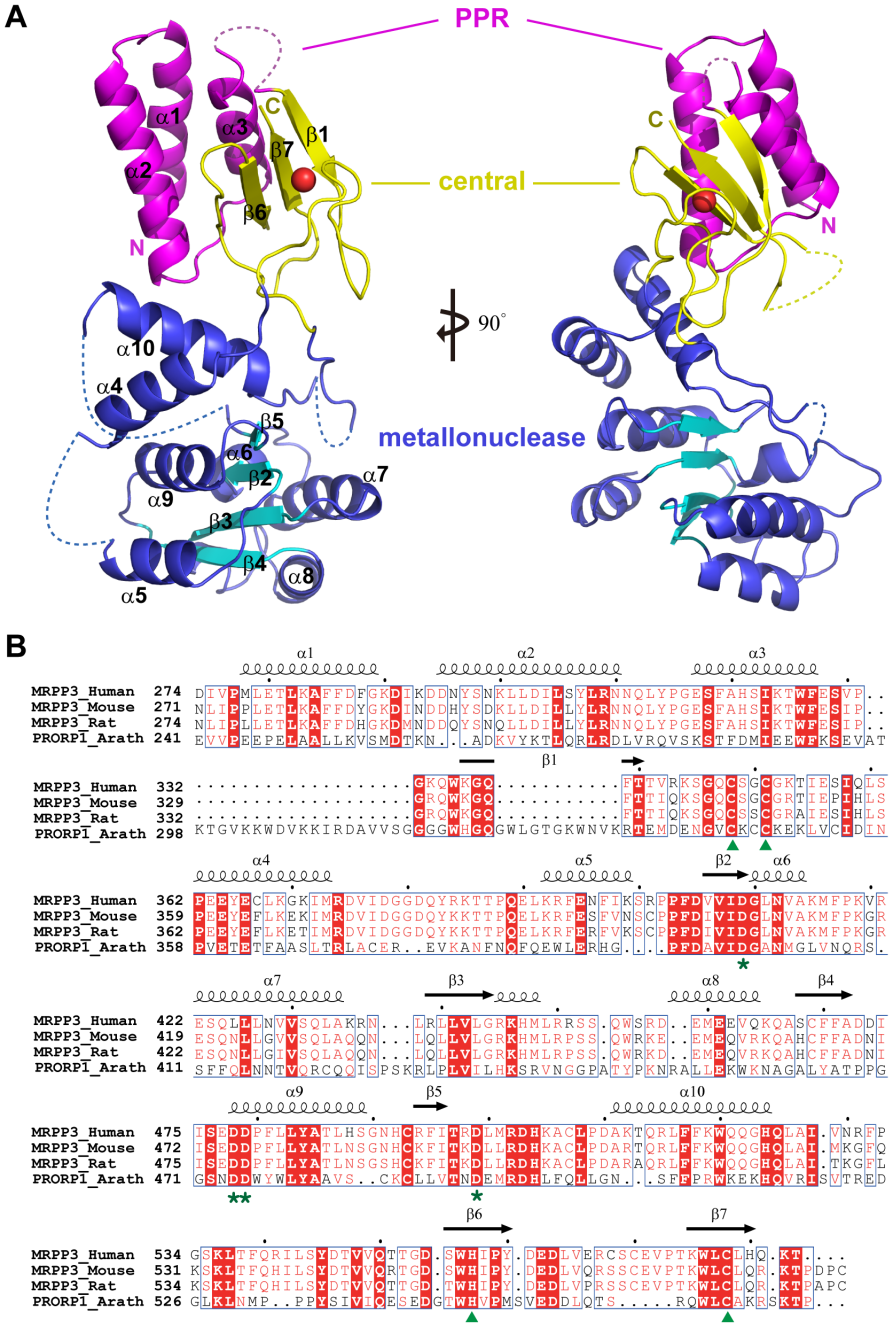
Overall structure of MRPP3-C. (A) Cartoon representation of the overall structure of MRPP3-C. The PPR domain, the center domain and the metallonuclease are colored magenta, yellow and blue, respectively. The zinc atom is represented as a red sphere. (B) Aligned amino acid sequences of the MRPP3 protein from *Homo sapiens, Mus musculus* and *Rattus norvegicus*, as well as the PRORP1 protein from *Arabidopsis thaliana*. The residues that are conserved among the species are highlighted in red. The secondary structure is drawn based on the human MRPP3-C structure. The CCCH Zn-binding site is designated with green arrowheads, and the active site is designated with green stars.

The crystal structure of MRPP3-C was solved by single-wavelength anomalous dispersion methodology and refined to a resolution of 2.7 Å. The overall structure consists of three domains (Figure 1A): the PPR domain (residues 274–331), the central domain (residues 332–359 and residues 536–583) and the metallonuclease domain (residues 360–535). The PPR domain contains three α-helices corresponding to helices α9, α10 and α11 in the structure of *Arabidopsis thaliana* PRORP1^8^. The PPR domain has been implicated as interacting with single-stranded RNAs in a sequence-specific manner^24^,^25^. Thus, we speculated that the PPR domain in the MRPP3 enzyme may interact with the single-stranded 5′ leader of pre-tRNA.

The central domain links the PPR domain and the metallonuclease domain. This domain contains a zinc-binding motif and consists of three antiparallel stranded β-sheets with some long flexible loops (Figure 1A). The zinc binding sites are highly conserved across different species (Figure 1B): residues C348 and C351 are located on the loop between β1 and α4, whereas residues H557 and C578 are separately located at β6 and β7. CCHC coordination with zinc ions fixes the loop together with β6 and β7 and stabilizes the entire central domain. The CCHC zinc motif may play a role in nucleotide binding.

The metallonuclease domain is located at the bottom of the entire structure. The inner core consists of four parallel β-sheets that are surrounded by seven α-helices (Figure 1A). No Mg^2+^ was found in the structure of the metallonuclease domain of MRPP3-C, although the crystallization conditions included 200 mM MgCl_2_.

**Figure 2:**
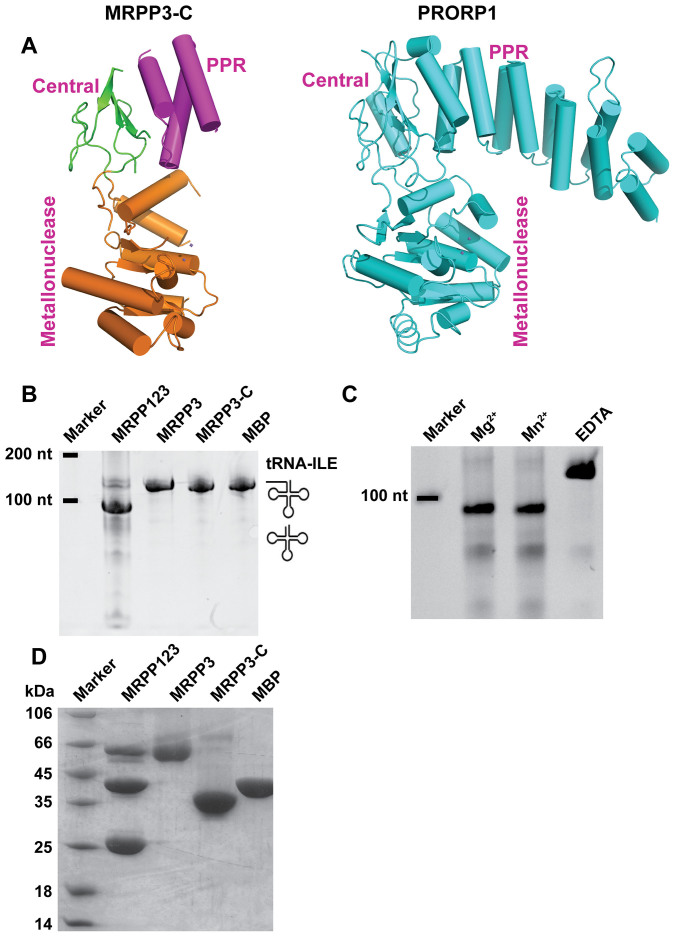
MRPP3 is inactive. (A) Ribbon representation of the overall structure of MRPP3-C and PRORP1. For MRPP3-C, the PPR domain, the center domain and the metallonuclease domain are colored purple, green and gold, respectively. (B) *in vitro* cleavage assays with the human mitochondrial tRNA^Ile^ precursor. (C) *in vitro* assays with 5 mM Mg^2+^, Mn^2+^ or EDTA. (D) Purity of the proteins used in the *in vitro* cleavage assays. Full-length gels are presented in Supplementary Figure S2.

The overall topology of MRPP3-C is similar to that of *Arabidopsis thaliana* PRORP1 (Figure 2A). The superposition of both structures gives a root-mean-square deviation (r.m.s.d.) of 2.93 Å for a total of 216 Cα atoms.

### Auto-inhibitory conformation of the metallonuclease domain of MRPP3-C

As mentioned above, PRORP1 and MRPP3 have similar structural topology but the organization of the active sites differ^26^,^27^. To further confirm this point, we examined the RNase P-like activity of purified MRPP3-C and full-length MRPP3 proteins. Human mitochondrial pre-tRNA^Ile^ was used as a substrate in the activity assay. The results showed that only the MRPP123 complex was able to correctly remove the 5′ leader of human mitochondrial pre-tRNA^Ile^. Neither MRPP3-C nor full-length MRPP3 showed any activity (Figure 2B and 2D, Supplementary Figure S1A and S2).

**Figure 3:**
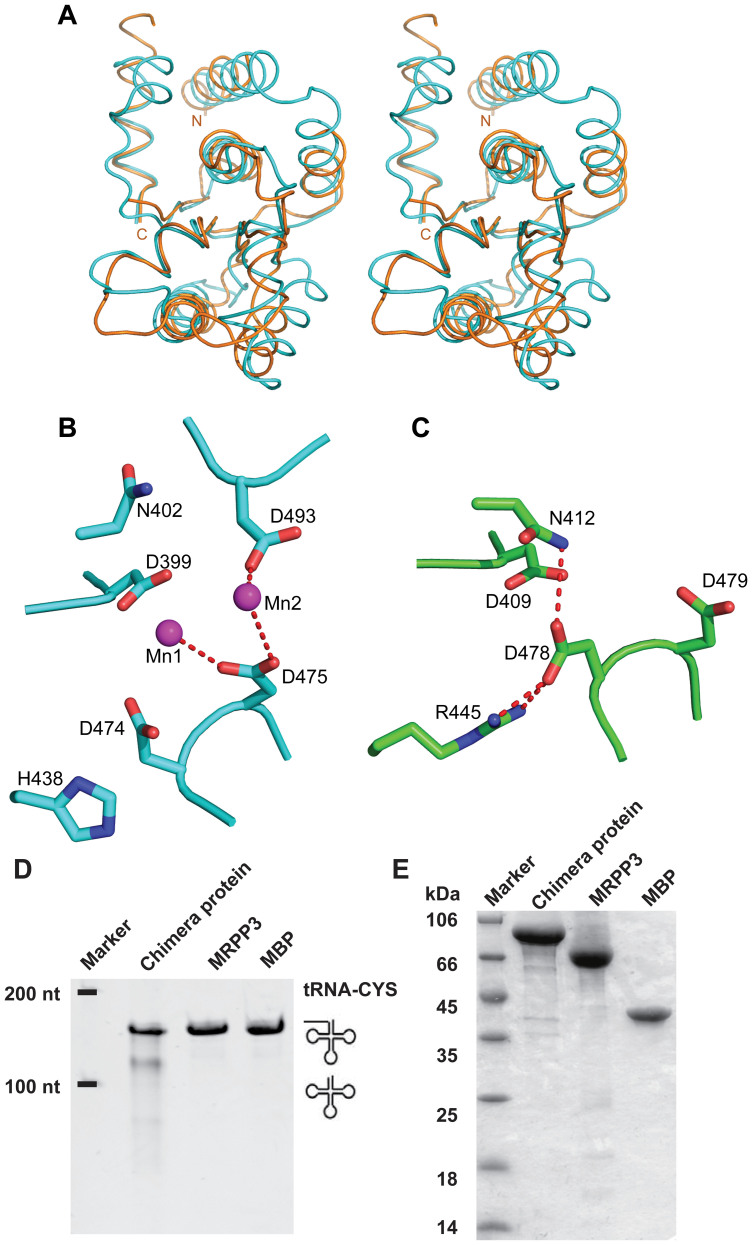
Auto-inhibitory conformation of the active site of MRPP3. (A) Stereo view of a structural comparison between the metallonuclease domains of MRPP3-C and PRORP1. The metallonuclease domain of MRPP3-C is colored gold and the metallonuclease domain of PRORP1 is colored cyan. (B) The catalytic center of PRORP1. Two Mn^2+^ ions (purple) are coordinated by residues D475 and D493. (C) The catalytic center of MRPP3-C. The red dashed lines indicate the salt bridge between the side chains of D478 and R445 as well as the hydrogen bond between D478 and N412. The oxygen and nitrogen atoms are colored red and blue, respectively. (D) *in vitro* cleavage assays with the *Arabidopsis thaliana* mitochondrial tRNA^Cys^ precursor. (E) Purity of the proteins used in the *in vitro* cleavage assays. Full-length gels are presented in Supplementary Figure S2.

To ascertain why MRPP3 is unable to carry out the catalytic activity alone, we first compared the metallonuclease domains of MRPP3-C and PRORP1 in detail (Figure 3A). The superposition of the two metallonuclease domains gave an r.m.s.d. value of 2.0 Å for 125 Cα atoms, indicating similar topology between these domains. We then compared key amino acids between these two domains. It has been proposed that both endoribonuclease RNase P and PRORP1 catalyze RNA cleavage using a two-metal-ion mechanism^28^. We confirmed that the MRPP123 complex also requires metal ions for activity (Figure 2C and 2D, Supplementary Figure S1B and S2). Next, the key residues for coordinating metal ions were examined. Several residues (D399, D474, D475 and D493) in PRORP1 have been proposed to coordinate metal ions^8^,^16^. Corresponding residues (D409, D478, D479 and D499) were found in MRPP3-C. This result suggests that the catalytic activity differences are not due to the absence of key amino acids.

There is a possibility that the sequence diversity causes a local conformational change and induces a different catalytic pocket. Indeed, we found obvious differences in the catalytic pockets of the two structures. Two metals ions are directly coordinated by D475 and D493 in PRORP1 (Figure 3B), whereas no metal ions are found in MRPP3-C. In the catalytic pocket of MRPP3-C, the side chain of D478 makes a salt bridge with R445 and has an additional hydrogen bond with N412 (Figure 3C). This type of configuration fully blocks the first metal ion binding site. This result is in agreement with that no metal ions are observed in the catalytic pocket of MRPP3-C, although the crystallization solution contained a high concentration of Mg^2+^ (200 mM). In the structure of PRORP1, H438 corresponds to R445 in MRPP3. The shorter side chain of histidine rather than arginine frees the neighboring D474 residue, consequently providing enough space to accommodate metal ions in the catalytic pocket of PRORP1. Therefore, the arrangement of the catalytic pocket in MRPP3 prevents loading Mg^2+^, suggesting that the structure of the metallonuclease domain, or the whole MRPP3 protein, is in an auto-inhibitory state.

### An active chimeric protein

To test our hypothesis that the catalytic domain of MRPP3 is auto-inhibited, we generated and purified several mutants: single point mutations of Arg445Ala, Arg445His and Arg445Leu, which were intended to break the salt bridge between Arg445 and Asp478. Unexpectedly, these mutants were unstable and easily degraded, perhaps due to mutation-induced conformational collapse.

We therefore generated a chimeric protein in which the MRPP3 catalytic core (residues 360–535) was replaced by the PRORP1 catalytic core (residues 356–527). As expected, the chimeric protein showed catalytic activity when using the *Arabidopsis thaliana* mitochondrial tRNA^Cys^ precursor as a substrate (Figure 3D and 3E, Supplementary Figure S1C and S2), although it was not as efficient as the MRPP123 complex (Figure 2B) or wild-type PRORP1^8^. This result is in agreement with the model that the catalytic core of MRPP3 exists in an auto-inhibitory conformation.

## Discussion

The human mitochondrial RNase P-like MRPP123 complex is unique in that it contains three proteins^18^. MRPP3 is the catalytic protein, and the function of the other two proteins (MRPP1 and MRPP2) relative to RNase P-like activity remains unknown. This behavior is strikingly different from the bacterial RNase P and that of the eukaryotic nucleus, which consist of a catalytic RNA associated with one to ten proteins^6^,^10^. Additionally, this complex differs from the proteinaceous PRORPs found in plants, which consists of one polypeptide^18^.

In this study, we determined the crystal structure of MRPP3-C, a catalytic portion of the MRPP3 protein. As expected, its overall structural topology is similar to that of PRORP1, especially in the metallonuclease domain. Further structural comparison of both catalytic pockets showed that no metal ions were found in MRPP3-C, although the key amino acids in the catalytic pocket in the two proteins are nearly identical. Within the catalytic pocket of MRPP3-C, the side chain of D478 forms a salt bridge with R445 and a hydrogen bond with N412. Thus, the arrangement of the catalytic pocket in MRPP3-C is unfavorable for Mg^2+^ binding, strongly suggesting that MRPP3 itself is inactive in its resting state because cleavage depends on metal ions for catalysis. This model is in agreement with the result that the chimeric MRPP3 protein in which the catalytic domain is replaced by the corresponding part of PRORP1 partially restores the ability to catalyze the removal of the 5′ leader of the tRNA precursor. It is reasonable to hypothesize that additional regulation may be required for the activation of the MRPP3 protein.

MRPP1 is a methyltransferase, but methylation is not necessary for removal of the 5′ leader^20^,^29^. MRPP2 is a dehydrogenase; however, its dehydrogenase activity is not coupled with its role in endonucleation^20^,^21^. We speculate that formation of the MRPP123 complex unlocks the auto-inhibitory state of MRPP3. It is very common that RNases undergo a conformational change in RNA processing, especially in the conserved metal-binding site^1^,^30^. For example, the RNase H domain of PRP8 (pre-mRNA processing factor 8) undergoes a conformational rearrangement to allow Mg^2+^ coordination between two steps of RNA splicing^31^.

Human mitochondrial DNA is transcribed as precursor consecutive polycistronic transcripts, which primarily contain mt-mRNAs punctuated by tRNAs^1^,^32^,^33^. The cleavage of precursor tRNAs at both ends produces mature tRNA and mt-mRNA^26^,^32^. Furthermore, the abundance of mature RNAs seems to correlate with the generation of energy in mitochondria^32^,^34^. Therefore, the precise control of precursor tRNA cleavage is vital for mitochondrial function, which may explain why the human MRPP123 complex requires such delicate activity regulation. Our results revealed that the MRPP123 complex is auto-inhibited, providing a solid basis for further investigation of the molecular mechanism of MRPP123 complex activation.

## Methods

### Protein expression and purification

The full-length human MRPP3 gene (KIAA0391) was PCR amplified from HeLa cells. The N-terminal truncation (residues 274–583, MRPP-C) was PCR amplified from the full-length MRPP3 gene then cloned into the PET-32M vector, in which an N-terminal Trx tag was added and followed by a PreScission protease site. Construct correctness was confirmed by sequencing. The plasmids were then transformed into *Escherichia coli* BL21 (DE3) and over-expressed by induction with 0.2 mM isopropyl-1-thiogalactopyranoside (IPTG) at 25°C for 10 h.

The harvest cells were resuspended in buffer A (20 mM Tris-HCl, pH 7.5; 300 mM NaCl) and lysed using sonication. After centrifugation at 18,000 × *g* for 40 min, the supernatant was loaded onto Ni-NTA affinity columns and washed with buffer A. The column was separately washed with 5 columns of buffer B (20 mM Tris-HCl, pH 7.5; 300 mM NaCl and 10 mM imidazole) and 5 columns of buffer C (20 mM Tris-HCl, pH 7.5; 300 mM NaCl and 25 mM imidazole). The target protein was eluted using buffer D (20 mM Tris-HCl, pH 7.5; 300 mM NaCl and 300 mM imidazole) and cleaved with PreScission protease at 4°C for 16 h. The eluted protein solution was diluted with a buffer of 20 mM Tris-HCl, pH 7.5 to the concentration of 100 mM NaCl, after which it was loaded onto an SP cation-exchange column (GE Healthcare) and eluted with a NaCl gradient (100–1000 mM). The fractions of interest were pooled and loaded onto a Superdex200 gel-filtration column (GE Healthcare) with buffer E (20 mM Tris-HCl, pH 7.5; 300 mM NaCl and 5 mM MgCl_2_), and peak fractions were identified by SDS-PAGE, and concentrated using a Centricon device (Millipore).

The full-length human MRPP1 gene and MRPP2 gene were PCR amplified from HeLa cells. MRPP1 gene were cloned into the PET-28a vector, in which a C-terminal 6 × His tag was added, and MRPP2 gene was cloned into the PET-M vector, in which an N-terminal 6 × His tag was added. The plasmids were then co-transformed into *Escherichia coli* BL21 (DE3) and over-expressed by induction with 0.2 mM isopropyl-1-thiogalactopyranoside (IPTG) at 25°C for 12 h. The complex of MRPP1/MRPP2 was purified following the same protocol as used for the MRPP3 protein.

The chimera protein in which the MRPP3 catalytic core (residues 360–535) were replaced by the PRORP1 catalytic core (residues 356–527) gene was synthesized (GENEWIZ). And the gene was cloned into PET-MBP vector, in which an N-terminal MBP tag was added. The plasmids were then transformed into *Escherichia coli* BL21 (DE3) together and over-expressed by induction with 0.2 mM isopropyl-1-thiogalactopyranoside (IPTG) at 25°C for 10 h. The purification of the chimera protein was almost the same as that of the MRPP3 protein except that the MBP tag was retained.

The Se-Met derivative protein was produced following the same protocol as used for the wild-type protein, with the exception that methionine auxotroph *E. coli* B834 (DE3) cells and minimal medium were used to express the recombinant protein.

### Crystallization and data collection

The MRPP3-C protein was crystallized by combining 1 μl of protein solution (20 mg/ml; 20 mM Tris-HCl, pH 7.5; 300 mM NaCl, 5 mM MgCl_2_ and 0.5 mM TCEP) with an equal volume of a reservoir solution containing 0.2 M MgCl_2,_ 0.1 M HEPES pH 7.5 and 25% PEG3350. Crystals were obtained at 20°C by the sitting-drop vapor-diffusion method and were flash frozen in liquid N_2_ with a cryoprotectant (0.2 M MgCl_2,_ 0.1 M HEPES pH 7.5 and 35% PEG3350).

The wild-type data were collected on a Shanghai Synchrotron Radiation Facility BL17U1 beam line, and all data were processed using the HKL2000 software^35^. Single wavelength anomalous data were collected for Se-Met substituted crystals at element SE peak wavelength on a beam line BL-17A at the Photon Factory.

### Structure determination and refinement

The structure of MRPP3-C was determined by SAD methodology. The initial phases were then calculated using PHENIX Autosol^36^. The models were built using the COOT program^37^. After the initial model was built, iterative refinement was performed with the PHENIX refinement program and COOT. The orientations of the amino acid side-chains and bound water molecules were modeled on the basis of 2***F***_obs_ − ***F***_calc_ and ***F***_obs_ − ***F***_calc_ difference Fourier maps. The final structure had an ***R***-work value of 23.85% and an ***R***-free value of 27.77%. Detailed data collection and refinement statistics are summarized in Table 1. All of the figures were made by the program PyMol (DeLano Scientific LLC.).

### RNase P-like cleavage assays

The human mitochondrial pre-tRNA^Ile^ gene, which contains the T7 promoter, was synthesized (Genewiz). The template for mitochondrial pre-tRNA^Ile^ contains nucleotides 4235 to 4350 of the human mitochondrial genome cloned into the pUC57 vector. Linearization with *Bam*HI and *in vitro* transcription with T7 RNA polymerase resulted in a pre-tRNA containing a 47-nucleotide leader sequence and 24 nucleotides of a 3′-trailer sequence. The *Arabidopsis thaliana* mitochondrial pre-tRNA^Cys^ gene containing the T7 promoter was also synthesized (Genewiz). The entire pre-tRNA^Cys^ contains 161 nucleotides, including a 34-nucleotide leader sequence and 16 nucleotides long 3′-trailer.

The substrates were purified with a quick Oligo Purification Kit (Tiangen). Pre-tRNA processing reactions were carried out in 30 mM Tris-HCl pH 8.0, 40 mM NaCl, 4.5 mM MgCl_2_, 2 mM DTT and 100 units/ml RNasin (Takara). The assays were carried out in a volume of 20 µl, started with the addition of an appropriate amount of enzyme, incubated at 21°C for 10 hours, and stopped by adding EDTA. The cleavage products were separated by denaturing urea polyacrylamide gel electrophoresis and then soaked in ethidium bromide solution.

To perform the chemiluminescent nucleic acid detection experiment, the 3′ terminus of both pre-tRNA^Ile^ and pre-tRNA^Cys^ was ligated with a single biotinylated nucleotide using a Pierce RNA 3′ End Biotinylation Kit (Thermo Scientific). The substrates were then purified with a quick Oligo Purification Kit (Tiangen). The cleavage experiment was performed as described above except that the reaction time was changed to 5 hours. The cleavage products were separated by denaturing urea polyacrylamide gel electrophoresis and then transferred onto Biodyne B nylon membranes (Thermo Scientific) at 500 mA for 30 minutes. The transferred RNA products were crosslinked to the nylon membrane by exposing it to UV light for 10 minutes. The results were detected with a Chemiluminescent Nucleic Acid Detection Module Kit (Thermo Scientific).

## Author Contributions

F.L. and X.L. did protein purification; F.L. did crystallization, structure refinement and biochemical experiments; X.Y. did data collection and structure determination; W.Z. analyzed the data. F.L. and Y.S. designed the study and wrote the paper. All authors discussed the results and commented on the manuscript.

## Supplementary Material

Supplementary Information Supplemental Information

## Figures and Tables

**Table 1:** Data collection and refinement statistics

Crystal name	Wild-type-crystal	Se-Met-crystal
Unit cell		
Space group	***P***2_1_2_1_2_1_	***P***2_1_2_1_2_1_
***A***, ***b***, ***c*** (Å)	***a*** = 66.434; ***b*** = 78.287; ***c*** = 114.187	***a*** = 68.293; ***b*** = 80.888; ***c*** = 116.558
***Α***, ***β***, ***γ*** (°)	***α*** = ***β*** = ***γ*** = 90.00	***α*** = ***β*** = ***γ*** = 90.00
Molecule/asu	2	2
Wavelength (Å)	0.9791	0.9791
Resolution range (Å)	50−2.7 (2.8−2.7)	50−2.9 (3.00−2. 9)
No. of unique reflections	18,642	14,858
Redundancy	3.8(3.7)^a^	14.3(14.5)^a^
***R***_sym_ (%)^b^	6.2(29.6)^a^	8.0 (70.6)^a^
***I***/***σ***	20.03 (8.00)^a^	47.00(4.20)^a^
Completeness (%)	98.64 (97.01)^a^	97.4 (83.9)^a^
Refinement		
Resolution range (Å)	46.1 ~ 2.7	
***R***_crystal_ (%)^c^	23.85 (29.88)	
***R***_free_ (%)^d^	27.77 (37.03)	
RMSD_bond_ (Å)	0.005	
RMSD_angle_(°)	0.94	
Number of non-hydrogen atoms	3902	
macromolecules	3888	
protein residues	503	
Ligands	5	
Water	9	
Residues in (%)		
Ramachandran favored	97	
Ramachandran allowed	3	
Ramachandran outliers	0	
Clashscore	8.42	
Average B factor (Å^2^) of		
Chain A	69.310	
Chain B	69.310	
Solvent	68.203	

athe highest resolution shell;

b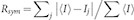;

c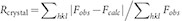;

d***R***_free_, calculated the same as ***R***_crystal_, but from a test set containing 5% of data excluded from the refinement calculation.
