# Supplementary material for: Auto-inhibitory Mechanism of the Human Mitochondrial RNase P Protein Complex
Source: Sci Rep. 2015 Apr 30;5:9878. doi: 10.1038/srep09878 (PMC4415599; doi:10.1038/srep09878)
Supplement: Supplementary Information — Supplemental Information [file srep09878-s1.pdf]

Supplementary Information

Auto-inhibitory Mechanism of the Human Mitochondrial RNase P Protein Complex

Fengzhi Li, Xiaofen Liu, Weihong Zhou, Xue Yang, Yuequan Shen

State Key Laboratory of Medicinal Chemical Biology, Nankai University, 94 Weijin Road, Tianjin 300071, China

Supplemental Figure 1

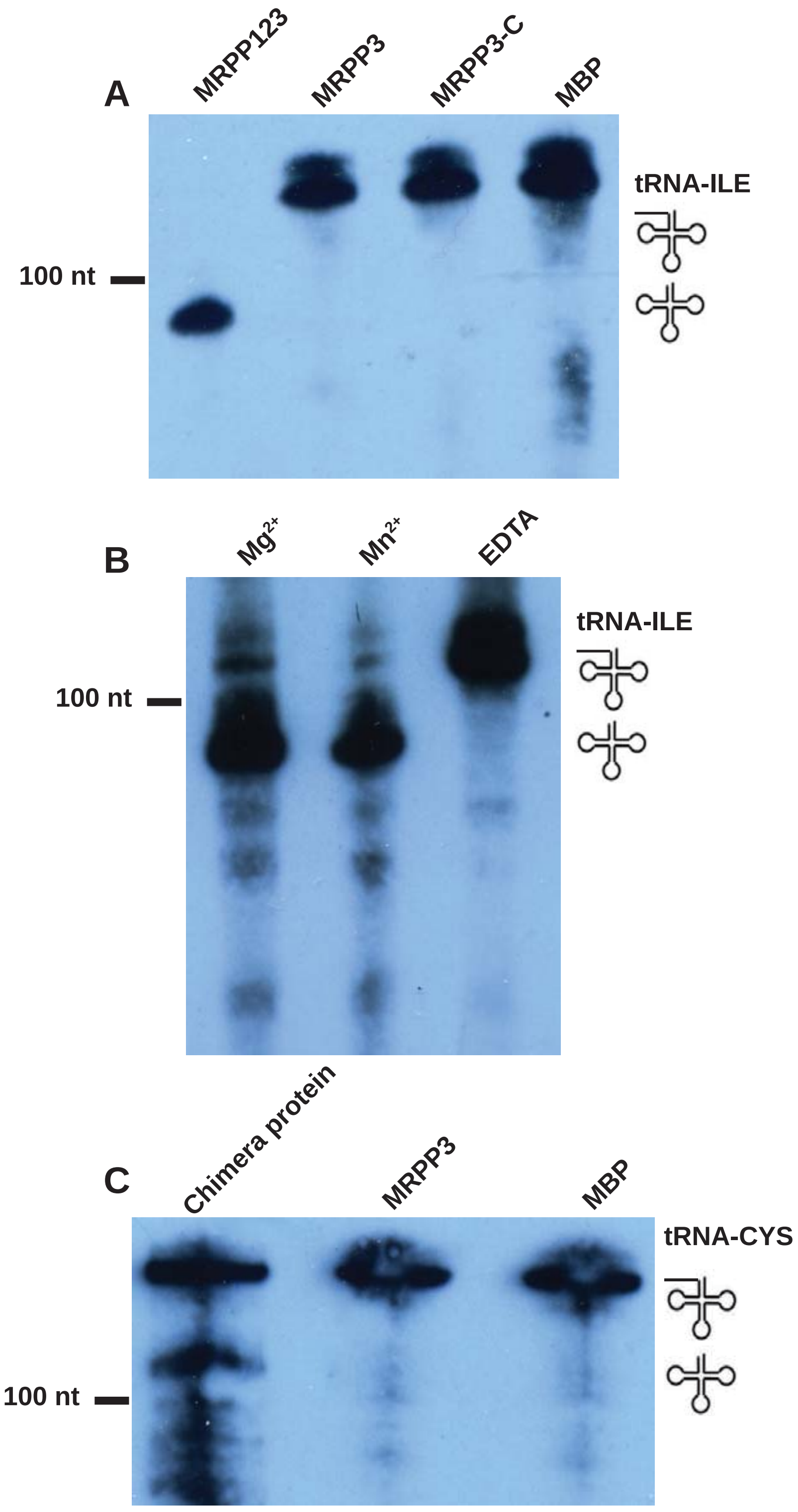

RNase P-like cleavage assays with chemiluminescent nucleic acid detection. (A), In vitro cleavage assay with biotinylated human mitochondrial $tRNA^{Ile}$ precursor at the 3' terminus. 5 μg of protein was used in the assay. (B), In vitro assays in the presence of Mg^{2+}, Mn^{2+} or EDTA. MRPP123 complex (5 μg) and human mitochondrial $tRNA^{Ile}$ precursor were used in the assay. (C), In vitro cleavage assays with biotinylated *Arabidopsis thaliana* $tRNA^{Cys}$ precursor at the 3' terminus. 5 μg of protein was used in the assay.

Supplemental Figure 2

Full-length Gels

for Figure 2B

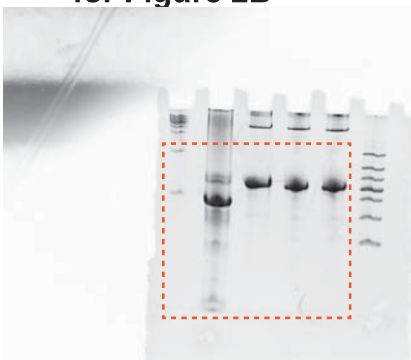

for Figure 2C

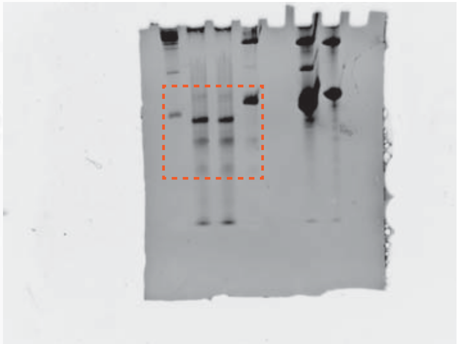

for Figure 2D

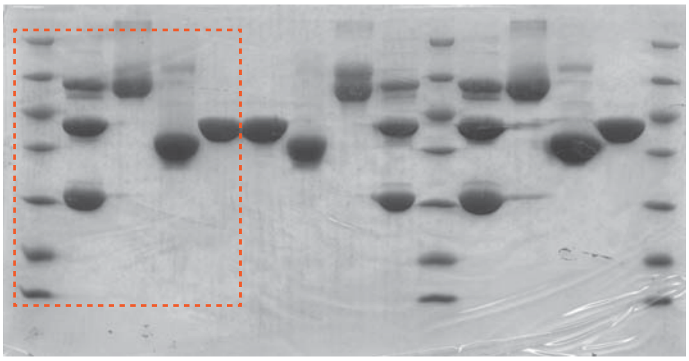

for Figure 3D

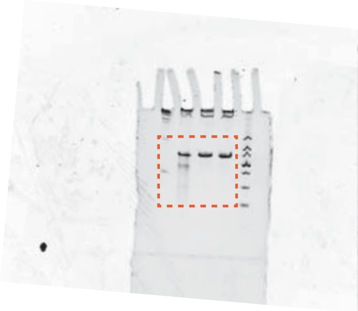

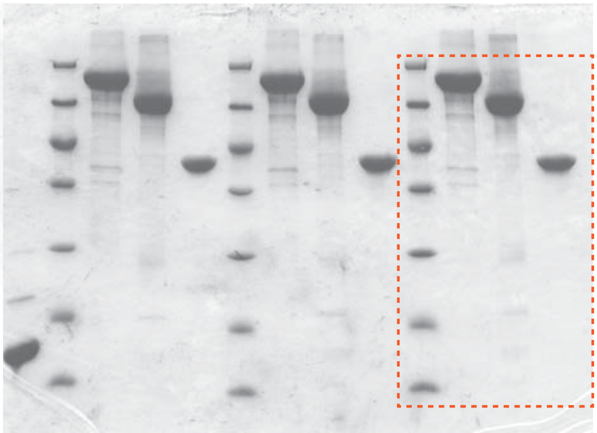

for Figure 3E

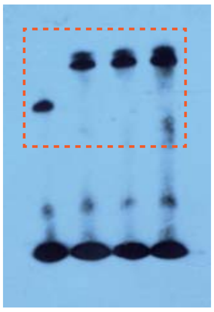

for Supplemental Figure S1A

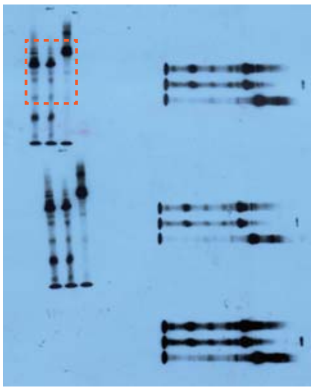

for Supplemental Figure S1B

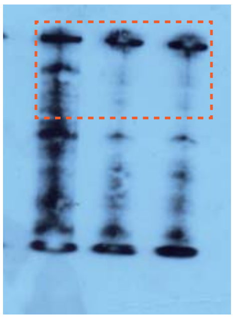

for Supplemental Figure S1C
